# Hand hygiene among oral health care providers in public institutions in Edo state even in the wake of Lassa fever

**DOI:** 10.4314/ahs.v23i4.21

**Published:** 2023-12

**Authors:** Omokhua Harrison, Enabulele Joan

**Affiliations:** University of Benin, Restorative Dentistry

**Keywords:** Hand hygiene, oral health care providers, public institution

## Abstract

**Background:**

Hand hygiene is recognized as the leading measure to prevent the cross-transmission of microorganisms and to reduce the incidence of healthcare-associated infections.

**Objectives:**

To assess the knowledge and practice of hygiene among dental health workers even in the wake of Lassa fever.

**Method:**

This questionnaire-based descriptive cross-sectional study was carried out in four public hospitals in Edo state. All data were collected using a self-administered structured questionnaire after obtaining ethical clearance.

**Results:**

108 of the 120 questionnaires distributed were filled and returned giving a response rate of 90.0%. Overall assessment of respondents' knowledge of hand hygiene revealed that 41.7% of the respondents possessed a good knowledge of hand hygiene, 37.0% had excellent knowledge, 17.6% had moderate and 3.7% had a weak knowledge of hand hygiene. Overall assessment of the practice of hand hygiene showed that the hand hygiene practice of 58.3% of respondents was fair while 37.0% practiced hand hygiene poorly. Only a few (5.6%) respondents had good hand hygiene practices.

**Conclusion:**

The outbreak of Lassa fever does not seem to positively influence the practice of hand hygiene among the respondents. While knowledge of hand hygiene is satisfactory in this study, the practice still leaves much to be desired.

## Introduction

Lassa hemorrhagic fever (LHF) is an acute viral hemorrhagic fever caused by the Lassa virus and was first described in 1969 in the town of Lassa where the first case of Lassa fever was reported, in Borno State, Nigeria.^1^ Lassa virus belongs to a member of the *Arenaviridae* virus family. Similar to Ebola Virus disease,^2^ clinical cases of Lassa fever had been discovered over ten (10) years, but had not been connected with a viral pathogen. LHF is often seen in people of West Africa. Outbreaks of the disease have been observed in Nigeria, Liberia, Sierra Leone, Guinea, and the Republic of Congo. Very recently there was an outbreak in Nigeria during which more than 10 states, including Edo state, were affected. Apart from rodent control measures, effective personal hygiene, especially hand hygiene plays an important role in the prevention of the spread of Lassa fever.

Hand hygiene is recognized as the leading measure to prevent the cross-transmission of microorganisms and to reduce the incidence of healthcare-associated infections.^3^,^4^ Despite the relative simplicity of this procedure, compliance with hand hygiene among healthcare providers is as low as 40%.^5^,^6^ In order to address this problem, continuous efforts are being made to identify effective and sustainable strategies. One such effort is the introduction of an evidence-based concept of “My five moments for hand hygiene” by the World Health Organization.^7^ These five moments call, for the use of hand hygiene include the moment before touching a patient, before performing aseptic and clean procedures, after being at risk of exposure to body fluids, after touching a patient, and after touching patient surroundings. This concept has been aptly used to improve understanding, training, monitoring, and reporting hand hygiene among healthcare workers.^3^

The hands of health care workers have been reportedly to be the most common carriers of transmission of microorganisms from one patient to another, one part of the patient's body to another as well as from the environment to the patient.^8^ Observing hand hygiene by all health care providers can be an essential step in preventing spread of infections from one person to another as well as to patients.

An outbreak of Lassa fever occurred in Edo state and neighbouring states with health workers getting infected in the line of duty. It is expected that with an outbreak of Lassa fever health care workers in general will be cautious of modes of transmission of this disease thereby curbing its spread. Hand hygiene is very important in disrupting the spread of Lassa hemorrhagic fever. This study therefore becomes important in the wake of LHF to assess the level of knowledge of hand hygiene as well as hand hygiene compliance and barriers to its effective practice among oral healthcare workers.

## Materials and method

This questionnaire based cross-sectional descriptive study was carried out in four public hospitals in Edo state. The study population was the entire dental health workers in the hospitals. All data were collected after obtaining Ethical clearance from the ethics and Research committee of the university of Benin, Benin city.

Data was collected using self-administered structured questionnaires. The questionnaires consisted of five sections. Section A elicited information on sociodemographic characteristics and profession of the study participants, Section B consisted of questions about knowledge of infection control, Section C, knowledge of hand washing, section D, practice of hand washing and section E barriers to effective hand washing. Information collected from the respondents were analysed with IBM SPSS version 21.0 software. Descriptive statistics, frequencies, tables and charts were used to summarize variables of interest while association between different variables were tested using chi-square test with p-value set at 0.05.

Assessment of knowledge of handwashing was done by awarding every correct response a score of 1 and incorrect response a score of 0. The highest possible score obtainable was 17 and the lowest possible score obtainable was 0. All scores obtained were summed up and converted to percentage which was graded using formats reported in previous studies9,10 as follows: <25%-Weak knowledge, 25-50%-Moderate knowledge, 51-75%-Good knowledge and >75%-Excellent knowledge.

Assessment of practice of handwashing was done by awarding every correct response a score of 1 and incorrect response a score of 0. The highest possible score obtainable was 12 and the lowest possible score obtainable was 0. All scores obtained were summed up and converted to percentage which was graded using formats reported in previous studies;9,10

Scoring and grading of practice:11 Less than or equal to 50%=Poor practice 51-69%=Fair practice 70% and above=Good practice.

## Results

One hundred and eight of the 120 questionnaires distributed were filled and returned giving a response rate of 90.0%. There were more female respondents (54.6%) compared to male respondents (45.4%) giving a female to male ratio of 1.2:1. The respondents' age ranged from 18 to54 years with a mean age of 32.69±8.77years. With regards to the tribe of respondents, the Benis recorded the highest (29.6%) number of respondents. Dentists and dental students were the most represented oral health professionals among the respondents with more than two-thirds (63.9%) of the study population. (Table 1

**Table 1:** Sociodemographic Characteristics of the respondents

Characteristics	Frequency	Percentage
**Age group (years)**		
18-21	7	6.5
22-30	32	29.6
31-40	46	42.6
41-50	21	19.4
51-60	2	1.9

**Gender**		
Male	49	45.4
Female	59	54.6

**Tribe**		
Ibo	23	21.3
Yoruba	22	20.4
Esan	26	24.1
Urhobo	5	4.6
Benin	32	29.6

**Profession**		
Dental Surgery Assistant	16	14.8
Dental Surgery Assistant Student	10	9.3
Dentist	63	58.3
Dental technologist	8	7.4
Dental Laboratory assistant	2	1.9
Dental student	9	8.3

Total	108	100.0

Professional group has been expunged from the above table.

Table 2 shows the knowledge of source of infection, Germs already present within the patients' hands (46.3%) was the most frequent source of infection responsible for oral health care infection with only 7.4% of the respondents believing hospital air was the most frequent source of infection responsible for oral healthcare infection.

**Table 2:** Knowledge of infection source

	Frequency	Percent
**Most frequent source of germs responsible for infection**		
Hospital water	13	12.0
Hospital air	8	7.4
Germs present	50	46.3
Hospital surface	37	34.3

**Main route of cross transmission of infection between patients and healthcare provider**		
Healthcare workers hand not clean	50	46.3
Patients' exposure to colonized surface	3	2.8
Sharing non-invasive objects	47	43.5
Air circulation	8	7.4

**Minimum time needed for alcohol-based hand rub to kill most germs on your hands**		
20 sec	35	32.4
3 secs	9	8.3
1min	44	40.7
10 secs	20	18.5

**Have you had formal training in hand hygiene?**		
YES	57	52.8
NO	51	47.2

**Total.**	108	100.0

**Main route of cross transmission of infection from patients to healthcare worker.**	YES	NO
Needle	85(78.7)	6(5.6)
Aerosol	82(75.9)	10(9.3)
Coughing	62(57.4)	14(13.0)
Sneezing	60(55.6)	15(13.9)
Hand shake	41(38.0)	34(31.5)

**Main route of cross transmission of infection from healthcare workers to patients**		
Needle	47(43.5)	30(27.8)
Aerosol	58(53.7)	20(18.5)
Coughing	62(57.4)	12(11.1)
Sneezing	65(60.2)	4(3.7)

**Which hand hygiene action prevents transmission of germs to patients**		
Wash hand before touching patient	103(95.4)	3(2.8)
Wash hands after exposure to fluids	88(81.5	7(6.5)
Wash after exposure to surrounding	75(69.4)	15(13.9)
Wash hands before a clean procedure	85(78.7)	10(9.3)

**Which hand hygiene action prevents transmission of germs to health worker**		
Wash hand after touching patient	101(93.5)	0(0.0)
Wash hands after exposure to fluids	86(79.6	5(4.6)
Wash after exposure to surrounding	78(72.2)	14(13.0)
Wash hands before a clean procedure	76(70.4)	12(11.1)

**Which of the following is associated with increased likelihood of colonization of hands with germs**		
Wearing jewellery	68(63.0)	22(20.4)
Damaged skin	93(86.1)	9(8.3)
Artificial fingernails	93(86.1)	8(7.4)
Regular use of hand cream	34(31.5)	42(38.9)

**Which of the following about alcohol-based hand rub and washing with soap are true?**	Yes	No
Rubbing better than washing in cleaning	36(33.3)	62(57.4)
Rubbing causes skin dryness than washing	53(49.1)	47(43.5)
Rubbing more effective than washing	30(27.8)	68(63.0)
Rubbing & washing done in sequence	74(68.5)	27(25.0)
Rubbing kills more germs than washing	35(32.4)	9(8.3)

**Which type of hand hygiene methods is required in the following situations.**		
Before setting up instrument	20(18.5)	68(63.0)
Before giving injection	22(20.4)	69(63.9)
After emptying sputum bowl	10(9.3)	85(78.7)
After removing exam gloves	13(12.0)	85(78.7)
After swabbing dental chair	16(14.8)	84(77.8)
After exposure to blood	12(11.1)	85(78.7)

With regards to the main route of cross transmission of infection between patients and healthcare providers, majority, 46.3% of the respondents felt unclean hands of the healthcare providers was the main route of cross transmission of infection between patients and healthcare providers. Only a few, 2.8%, were of the opinion that patients' exposure to colonized surface was the main route of cross transmission of infection.

The knowledge of the minimum time needed for alcohol-based hand rub to kill most germs on the hands was assessed. Most 44(40.7%) of the respondents believed rubbing of alcohol-based solution for one minute was enough time to kill the germs on the hands. However, an appreciable number 35(32.4%) felt 20 seconds was enough time. A very small proportion (8.3) believed 2 seconds was enough time. Fifty -seven percent of the respondents claimed they have had formal training on hand hygiene.

Fifty-seven of the respondents claimed to have had a formal training in Hand hygiene.

Table 2 also depicts the main routes of cross-transmission of infection from patients to healthcare providers and vice versa. All the routes were believed to be the main routes of transmission except for hand shake that was reported by less than half (38.0%) of the proportion of the respondents. However, majority of the respondents (78.7%) felt needle prick was the main route of transmission of transmission of infection.

With regards to prevention of transmission of germs to patients and to healthcare providers, all the hand hygiene actions were believed to prevent transmission of germs to patients by at least 69.4% of the respondents. Washing of hand before touching patients was thought to be the most effective hand hygiene action according to 95.4% of respondents while washing of hands after touching the patient was reported by more than two-third (93.5%) of respondents as the most effective hand hygiene action in preventing transmission of germs to healthcare providers from patients.

In respect of alcohol-based hand rub and washing with soap and their effects on germs, majority (68.5%) of the respondents said rubbing and washing is done the in sequence was most effective against germs. More than half (63.0%) of the respondents were of the opinion that rubbing was not more effective than washing. Similarly, more than half of the respondents did not also feel that rubbing was better than washing and cleaning. A large percentage (40.7) claimed they did not know if rubbing killed germs more than washing compared to 32.4% who felt rubbing killed more germs.

Wearing of jewellery, damaged skin, and artificial fingernails were reported by at least more than half of the respondents as being closely associated with an increased likelihood of colonization of hands with germs.

Regarding the particular hand hygiene methods required in certain situations, it was observed that hand washing was the most preferred hand hygiene method before setting up instrument, before giving injection, after emptying the sputum bowl, after removing exam gloves, after swabbing the dental chair and after exposure to blood.

Concerning practice of hand hygiene by respondents, it was observed from table 4, that at least 78.7% of the respondents washed their hands after touching a patient, before touching a patient, after washing instrument and after swabbing the dental chair. Most (93.5%) of the respondents claimed they washed their hands with soap. Only 13.9% of the respondents agreed that they washed their hands without soap. More than two-thirds (73.1%) claimed they dried their hands with an electric hand drier while 65.7% and 72.2% denied drying their hands with tissue paper and on their clothes respectively. General use towel was the most commonly used hand towel by a majority (65.7%) of the respondents. The most popular type of tap system used by the respondents was manual with elbow (62.0%). Lack of soap was the most common barrier to effective hand hygiene reported by 65.7% of the respondents.

**Table 4:** Association between age group, gender, professional group and knowledge of hand hygiene

	Knowledge grade				Total	P value
	Weak n (%)	Moderate n (%)	Good n (%)	Excellent n (%)		
Age group (years) 18-21.	0(0.0)	2(28.6)	2(28.6)	3(42.9)	7(100.0)	0.801
22-30	0(0.0)	6(18.8)	15(46.9)	11(34.4)	32(100.0)	
31-40	2(4.3)	7(15.2)	21(45.7)	16(34.8)	46(100.0)	
41-50	2(9.5)	3(14.3)	7(33.3)	9(42.9)	21(100.0)	
51-60	0(0.0)	1(50.0)	0(0.0)	1(50.0)	2(100.0)	
Gender Male	4(8.2)	12(24.5)	16(32.7)	17(34.7)	49(100.0)	0.028
Female	0(0.0)	7(11.9)	29(49.2)	23(39.0)	59(100.0)	
Professional group DSA+DNS	0(0.0)	5(19.2)	11(42.3)	10(38.5)	26(100.0)	0.826
Dentist and Dental student	3(4.3)	13(18.8)	29(42.3)	24(34.8)	69(100.0)	
Dent technician and laboratory attendant	1(7.7)	1(7.7)	5(38.5)	6(46.2)	13(100.0)	
Total	4(3.7)	19(17.6)	45(41.7)	40(37.0)	108(100.0)	

• DSA= Dental surgery assistant; DNS = Dental nursing student

Overall assessment of the practice of hand hygiene showed that the hand hygiene practice of 58.3% of respondents was fair while 37.0% of respondents practiced hand hygiene poorly. Only very few (5.6%) respondents had good hand hygiene practices.

Table 4 represents the association between age group, gender, professional group and knowledge of hand hygiene. There was no statistically significant association between age group and the knowledge of hand hygiene(p>0.05). However, among the respondents in the age group 31-40 years, the majority (45.7%) had good knowledge of hand hygiene compared to 34.8% who recorded excellent knowledge. The best knowledge of hand hygiene among the respondents was recorded among those aged 31-40 years.

There was a statistically significant association between gender and knowledge of hand hygiene with female respondents recording better knowledge of hand hygiene. (p=0.028). More females (39.0%) had excellent knowledge of hand hygiene compared to males who had 34.7% excellent knowledge. More than half (49.2%) of female respondents had moderate knowledge while less than half (32.7%) of the male respondents had moderate knowledge.

The best knowledge of hand hygiene was observed among dentists and dental students. This was however not statistically significant(p<0.05). The majority (42.3%) of dentists and dental students had moderate knowledge. This was comparable to the 42.3% moderate knowledge also recorded by the DSA and DSA students. However, there were more than 24(34.8%) dentists and Dental students who had excellent knowledge of hand hygiene compared to 10(38.5%) DSA and DSA students

Table 5, the repeated Total has been undone and subunits separated by a blank row

**Table 5:** Association between age group, gender, professional group and practice of hand hygiene

	Practice grade			Total	p-value
	Poor n (%)	Fair n (%)	Good n (%)		
Age group (years) 18-21	1(14.3)	6(85.7)	0(0.0)	7(100.0)	0.703
22-30	15(46.9)	15(46.9)	2(6.3)	32(100.0)	
31-40	17(37.0)	27(58.7)	2(4.3)	46(100.0)	
41-50	7(33.3)	13(61.9)	1(4.8)	21(100.0)	
51-60	0(0.0)	2(100.0)	0(0.0)	2(100.0)	
Gender Male	30(61.2)	18(36.7)	1(2.0)	49(100.0)	**<0.0001**
Female	10(16.9)	45(76.3)	4(6.8)	59(100.0)	
Professional group DSA+DNS	5(19.2)	21(80.8)	0(0.0)	26(100.0)	0.105
Dentists and Dental students	30(43.5)	35(50.7)	4(5.8)	69(100.0)	
Dent technician and laboratory assistants	5(38.5)	7(53.4)	1(7.7)	13(100.0)	
Total	40(37.0)	63(58.3)	5(4.6)	108(100.0)	

• DSA= Dental surgery assistant; DNS = Dental nursing student

The association between age group, gender, professional groups of respondents and practice of hand hygiene is depicted in Table 5. There was a statistically significant association between gender and the practice of hand hygiene(p=0.000) indicating that gender affected the practice of hand hygiene among the respondents. More than two-thirds of the female respondents had a fair practice of hand hygiene compared to 36.7% of male respondents who had fair practice. Fewer persons, 6.8% of females and 2.0% of males had good practice of hand hygiene. The age group and professional groups of the respondents did not have any statistically significant association with hand hygiene practice (p>0.05). Very few respondents across the different age groups had good practices of hand hygiene. None (0.0%) of the respondents in the professional group, DSA and DSA students had good practice of hand hygiene.

## Discussion

Hand hygiene compliance among dental health workers is vital to effective infection control especially in the wake of Lassa fever. A thorough understanding of how microorganisms are transmitted between patients and dental health providers is crucial in ensuring efficient infection control in the dental practice space. Dentistry has a duty to observe scientifically accepted and evidenced-based principles of infection control.^12^

In this study, the majority of respondents had a good to excellent knowledge of hand hygiene. This high number is quite reassuring meaning that knowledge is not lacking. In this same study, most of the respondents recorded fair hand hygiene practices. This shows perhaps a lack of willpower to carry out hand hygiene practice by the respondents despite an encouraging knowledge of hand hygiene. It is expected that oral health providers will be more meticulous in the practice of hand hygiene in the wake of Lassa fever, especially as Edo state where the study was carried out is one of the states with high prevalence of Lassa fever and the state also houses a Lassa fever referral testing and treatment center. In a study carried out by Omogbai et al,^13^ the majority of the respondents had excellent knowledge of hand hygiene which is consistent with the present study. The presence of Lassa fever outbreak does not seem to have influenced hand hygiene practice positively in this research work.

Germs present (46.3%) was the most frequent source of oral health care infection in the dental practice according to this study while hospital air (7.4%) was perceived to be the least source of germs contamination by the respondents. Knowledge of sources of infections is vital in effective infection control. Healthcare workers' hands not being clean, sharing of non-invasive objects, use of needles, coughing, aerosol, sneezing and handshake have been identified by the respondents as routes of cross-transmission of infection from healthcare workers to patients and vice versa. This level of knowledge is encouraging as this may imply that many of the respondents will be willing to observe proper hand hygiene in their practice.

More than two-thirds, (95.4%) of the respondents agreed that washing hands before touching a patient was the correct order. Similarly, the majority of the participants were of the opinion that hand washing should also be carried out after exposure to fluids, and surrounding and before carrying out a clean procedure in order to prevent the spread of infections between healthcare workers and patients.

Knowledge about hand rubbing with alcohol-based agent and cleaning varied among the respondents. There was no consensus among the respondents on whether hand rubbing should be carried out before hand washing or vice versa.

Wearing jewellery, damaged skin, and fixing artificial nails were perceived by most respondents as being responsible for increased colonization of the hands by germs. This may be related to the fact these offer great reservoirs for organisms. Overall, the knowledge of respondents about hand hygiene was satisfactory, similar to reports by other studies.^12^,^14^

Hand hygiene in dental practice has been found to be one of the most important parts of the infection control process and is the single most important activity performed to reduce the risk of transmitting microorganisms from provider to patient. The microflora that inhabits the skin can be classified as transient or resident. Transient microflora colonizes the superficial layers of the skin and can be removed easily during routine hand washing. It also is the type of microflora that is transmitted most often when providing care directly to patients and is associated most frequently with healthcare-associated infections. Resident microflora is adherent and associated with the deeper layers of the skin, is most resistant to removal with HH, and is less likely to be associated with healthcare-associated infections. The selection of HH methods depends on factors such as the type of procedure to be performed, the persistence of decontamination, and the potential risk of spreading infection. Gloves, which often are thought to be a completely effective barrier that protects healthcare providers and prevents the spread of microorganisms, have microscopic imperfections.^12^ Hence, gloves can give providers a false sense of security. According to the CDC, the use of gloves reduces the risk of contamination by 70-80%, helps prevent cross-contamination, and helps protect patients and providers. Even though the use of gloves offers a means of protection, in addition, it creates a warm, moist environment in which organisms can proliferate.^12^

This situation results in a large increase in the amount of transient microflora. So, HH is essential to eliminate transient microflora and decrease resident microflora, even when gloves are worn. Data show that 51% of dental practitioners use soap and water for HH frequently and 44.6% use alcohol-based hand sanitizers for HH frequently, this is in agreement with CDC HH guidelines which recommend the use of alcohol-based hand sanitizer.^14^ However, this is not the case in the dental infection guidelines which place more importance on the use of soap and water.^14^ In addition, approximately one-third of the dental practitioners indicate that they have limited/moderate knowledge of the CDC HH guidelines.

Currently, the two most widely used antiseptics in use are alcohol-based rubs and medicated soaps or foams which contain chlorhexidine. Alcohols are effective against both gram-positive and gram-negative microorganisms. In this study, there were more respondents who believed hand washing is more important than hand rub. This is in keeping with a study by Myers et al which reported more subjects preferring hand washing to alcoholic rub.^14^

It was observed in this study that, more than two-thirds of the participants washed their hands either before touching patients, after touching patients, after washing instruments, or after swabbing the dental chair. This is consistent with the standard practice of HH. Electric hand driers and hand towels were the most commonly used means of hand drying after washing, with general-use towels and elbow control tap heads being the kind of towel and tap available. Lack of soap and adequate hand driers were seen as the most common barriers to effective hand hygiene practice according to the respondents in the present study. In a similar study by Sachin et al, a lack of soap was also reported as a barrier to hand washing.^12^

In the association between age group, gender, professional groups, and knowledge of HH, the age group 31-40 recorded excellent knowledge of HH. This may be due to the fact that those in this age group pay more attention to infection control, with HH as the primary means of controlling the infection. Gender had a statistically significant association, with female respondents having better knowledge. This may not be unconnected with the fact that females tend to be more hygiene conscious.

Similarly, female participants recorded better HH practice in the association between age group, professional group, gender, and HH practice, in which the association between HH and gender was statistically significant. This is maybe due to the fact that females are more hygiene conscious compared to males.

A review of a few of the available literature from previous studies on this subject matter shows only a marginal improvement in the knowledge and practice of HH. This is despite the increasing awareness of the importance of HH to both patients and health workers

## Conclusion

The outbreak of Lassa fever does not seem to positively influence the practice of hand hygiene among the respondents. While knowledge of hand hygiene is satisfactory in this study, the practice still leaves much to be desired. Dental healthcare providers need to be reminded that having good knowledge will not necessarily translate to better practice. Practical steps should be taken to encourage the increased practice of HH all the time. There should be objective measures of HH compliance among dental health providers with a view to making them imbibe the culture of HH at all times. Efforts should be made at removing the identified barriers to HH.

## Figures and Tables

**Figure 1:**
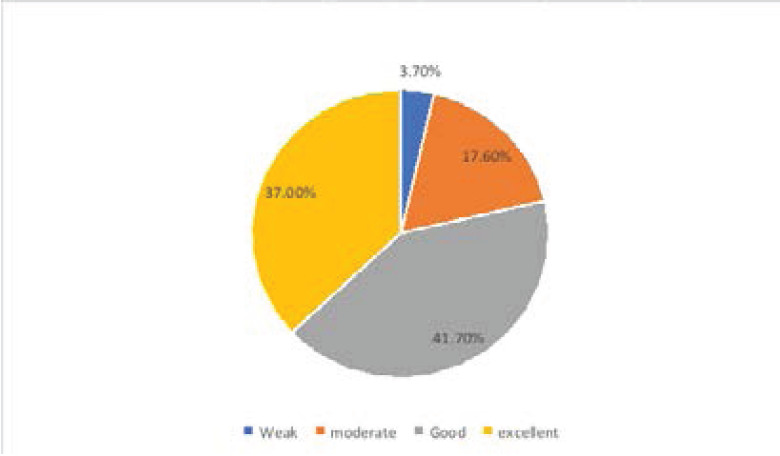
represent the grading of knowledge score by the respondents. Overall assessment of respondents' knowledge of hand hygiene revealed that, 41.7% of the respondents possessed good knowledge of hand hygiene, 37.0% excellent knowledge, 17.6% moderate and 3.7% had a weak knowledge of hand hygiene

**Table 3:** Practice of hand hygiene

**When do you wash your hands?**	Yes n (%)	No n (%)	Don't know n (%)	Total n (%)
After touching a patient	98(90.7)	8(7.4)	2(1.9)	108 (100.0)
Before touching patient	85(78.7)	13(12.0)	10(9.3)	108 (100.0)
After washing instrument	87(80.6)	11(10.2)	10(9.3)	108 (100.0)
After swabbing the dental chair	89(82.4)	12(11.1)	7(6.5)	108 (100.0)

**What do you wash your hands with**				
Soap	101(93.5)	5(4.6)	2(1.9)	108 (100.0)
Household bleach	26(24.1)	67(62.0)	15(13.9)	108 (100.0)
Without soap	15(13.9)	75(69.4)	18(16.7)	108 (100.0)
Methylated spirit	27(25.0)	69(63.9)	12(11.1)	108 (100.0)

**What do you dry your hands with?**				
Hand towel	65(60.2)	33(30.6)	10(9.3)	108 (100.0)
Electric hand drier	79(73.1)	27(25.0)	2(1.9)	108 (100.0)
Air drying	47(43.5)	48(44.4)	13(12.0)	108 (100.0)
Tissue paper	26(24.1)	71(65.7)	11(10.2)	108 (100.0)
Dry on your clothes	14(13.0)	78(72.2)	16(14.8)	108 (100.0)

**Kind of hand towel available in your center**				
Single use towel	24(22.2)	79(73.1)	5(4.6)	108 (100.0)
General use towel	71(65.7)	34(31.5)	3(2.8)	108 (100.0)
Disposable towel	30(27.8)	76(70.4)	2(1.9)	108 (100.0)

**What kind of tap system is available in your Centre**				
Manual with elbow control head	67(62.0)	39(36.1)	2(1.9)	108 (100.0)
Manual with finger control head	50(46.3)	58(53.7)	0(0.0)	108 (100.0)
Sensor control type	11(10.2)	95(88.0)	2(1.9)	108 (100.0)

**Barriers to effective hand washing.**				
Lack of water	64(59.3)	37(34.3)	7(6.5)	108 (100.0)
Lack of soap	71(65.7)	27(25.0)	10(9.3)	108 (100.0)
Lack of sink	29(26.9)	60(55.6)	19(17.6)	108 (100.0)
Lack of adequate hand driers	71(65.7)	31(28.7)	6(5.6)	108 (100.0)

**Figure 2:**
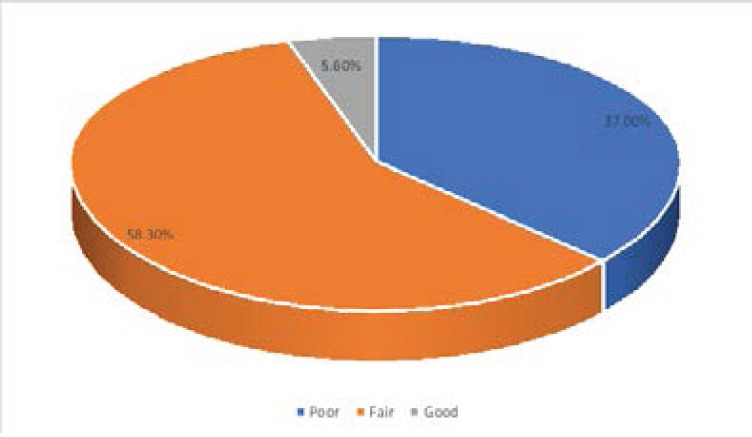
Grading of hand hygiene practice
